# *MGMT* Promoter Methylation as a Prognostic Factor in Primary Glioblastoma: A Single-Institution Observational Study

**DOI:** 10.3390/biomedicines10082030

**Published:** 2022-08-20

**Authors:** Mateusz Szylberg, Paweł Sokal, Paulina Śledzińska, Marek Bebyn, Stanisław Krajewski, Łukasz Szylberg, Aneta Szylberg, Tadeusz Szylberg, Kamil Krystkiewicz, Marcin Birski, Marek Harat, Robert Włodarski, Jacek Furtak

**Affiliations:** 1 Department of Neurosurgery and Neurology, Jan Biziel University Hospital Nr 2, Collegium Medicum, Nicolaus Copernicus University, 85-168 Bydgoszcz, Poland; 2 Department of Neurosurgery, 10th Military Research Hospital and Polyclinic, 85-681 Bydgoszcz, Poland; 3 Molecular Oncology and Genetics Department, Innovative Medical Forum, The F. Lukaszczyk Oncology Center, 85-796 Bydgoszcz, Poland; 4 Department of Physiotherapy, University of Bydgoszcz, Unii Lubelskiej 4, 85-059 Bydgoszcz, Poland; 5 Department of Pathology, 10th Military Research Hospital, and Polyclinic, 85-681 Bydgoszcz, Poland; 6 Department of Perinatology, Gynaecology and Gynaecologic Oncology, Collegium Medicum, Nicolaus Copernicus University in Bydgoszcz, 85-168 Bydgoszcz, Poland; 7 Department of Internal Diseases, 10th Military Research Hospital and Polyclinic, 85-681 Bydgoszcz, Poland; 8 Department of Neurosurgery and Neurooncology, Nicolaus Copernicus Memorial Hospital, 93-513 Lodz, Poland; 9 Department of Anaesthesiology and Intensive Care, 10th Military Research Hospital and Polyclinic, 85-681 Bydgoszcz, Poland; 10 Department of Neurooncology and Radiosurgery, Franciszek Łukaszczyk Oncology Center, 85-796 Bydgoszcz, Poland

**Keywords:** glioblastoma, *MGMT*, prognostic factor, survival prediction, surgical resection

## Abstract

Glioblastoma is the most malignant central nervous system tumor, which represents 50% of all glial tumors. The understanding of glioma genesis, prognostic evaluation, and treatment planning has been significantly enhanced by the discovery of molecular genetic biomarkers. This study aimed to evaluate survival in patients with primary glioblastoma concerning O6-methylguanine–DNA methyltransferase (*MGMT*) promoter methylation and other clinical factors. The study included 41 newly diagnosed glioblastoma patients treated from 2011 to 2014 in the 10th Military Research Hospital and Polyclinic, Poland. All patients underwent surgical resection followed by radiation and chemotherapy with alkylating agents. The *MGMT* promoter methylation was evaluated in all patients, and 43% were found to be methylated. In 26 and 15 cases, gross total resection and subtotal resection were conducted, respectively. Patients with a methylated *MGMT* promoter had a median survival of 504 days, while those without methylation had a median survival of 329 days. The group that was examined had a median age of 53. In a patient group younger than 53 years, those with methylation had significantly longer overall survival (639 days), compared to 433.5 days for patients without methylation. The most prolonged survival (551 days) was in patients with *MGMT* promoter methylation after gross total resection. The value of *MGMT* promoter methylation as a predictive biomarker is widely acknowledged. However, its prognostic significance remains unclear. Our findings proved that *MGMT* promoter methylation is also an essential positive prognostic biomarker.

## 1. Introduction

Glioblastoma multiforme (GBM) is a malignant tumor originating from glial cells. It is the most common primary brain tumor in adults, with a poor prognosis and a high mortality rate. Current standard treatment methods for newly diagnosed malignant glioma patients include maximum safe resection followed by a combination of radiation and chemotherapy, mostly with alkylating agents (temozolomide-TMZ). These methods frequently fail to achieve a satisfactory effect due to tumor heterogeneity and chemoresistance to cytotoxic alkylating agents. Despite rigorous therapy, the median survival rate for most patients is about 1 to 2 years after diagnosis [1,2]. Furthermore, GBM recurs in nearly all patients independently of the tumor stage at the time of surgery or the treatment methods employed. About ninety percent of recurrences are detected in the initial tumor site [3]. Numerous researchers have defined the methylation status of the *MGMT* gene promoter as a possible predictor of response to chemotherapy, particularly alkylating drugs such as temozolomide [4,5]. The *MGMT* gene is located on chromosome 10q26 and encodes a DNA-repair protein that removes alkyl groups from the O^6^ position of guanine, an essential DNA alkylation site. The *MGMT* gene is involved with DNA repair and in the resistance to alkylating drugs of glioma cells [6,7]. The DNA methylation induced by TMZ is located at the O^6^-position of guanine, and methylation at this position is considered the main contributor to the cytotoxic effect. TMZ alkylates the DNA of cells at all phases of the cell cycle, although S-phase progression is required; thus, it is S-phase dependent. TMZ induces mispairing of bases which is detected by the mismatch repair mechanism (MMR). This MMR processing and S-phase development results in double-stranded breaks. Therefore, MMR is essential for TMZ to exert its cytotoxic effect [8,9]. 

There is evidence that methylation of the *MGMT* promoter also occurs in histologically normal brain tissue. However, predisposition is caused by an epigenetically controlled deficiency in the *MGMT* gene and alkylation-related mutations are the primary driving force [10]. The epigenetic suppression of *MGMT* by hypermethylation of cytidine–phosphate–guanine dinucleotides (CpG islands) in the promoter region is responsible for the decreased *MGMT* activity in glioma cells [11]. Studies of GBM patients showed that hyper-methylation of the *MGMT* promoter correlates with improved overall survival (OS) and a more significant response to the TMZ treatment [12,13]. In glioma cells, the level of MGMT protein expression has been related to the efficacy of alkylating agents [14,15,16]. In addition, combined inactivation of *MGMT* by promoter methylation and deletion of 10q increases sensitivity to TMZ compared to promoter methylation or loss of 10q alone [17]. The best TMZ response rates were associated with *IDH* mutations in conjunction with *MGMT* promoter methylation [18]. Moreover, a previous study demonstrated that patients with an *IDH1* gene mutation and *MGMT* promoter methylation who underwent radiation had a more favorable prognosis compared to patients with methylation of *MGMT* promoter alone [19]. Although the positive predictive value of *MGMT* promoter methylation in glioblastoma patients is widely acknowledged, the prognostic value remains unclear or controversial [20,21,22,23,24,25].

The purpose of the present study was to assess the prognostic significance of *MGMT* promoter methylation and its associations with clinical variables.

## 2. Materials and Methods

The study included 41 newly diagnosed glioblastoma patients treated from 2011 to 2014 in the Department of Neurosurgery, the 10th Military Research Hospital and Polyclinic in Bydgoszcz, Poland. Baseline assessments consisted of physical and neurological examinations [26,27]. Before the surgical resection, the patient’s neurological state was evaluated and their Karnofsky Performance Status (KPS) was no lower than 80 points. Patients whose KPS scores are lower have a worsening functional level (100 points—no evidence of disease, no symptoms; 0 points—death) [26]. After taking into account the patient’s physical condition, as well as any comorbidities, the anesthesiologist classified each patient according to the guidelines established by the American Society of Anesthesiologists (ASA) (ASA score of 1—healthy person; ASA score of 2—mild systemic disease; ASA score of 3—severe systemic disease) [27]. All patients underwent surgical resection, followed by radiotherapy with alkylating-agent-based chemotherapy. Gross total resection (GTR) was defined as resection without a visual residual enhancing tumor. The exclusion criteria were the presence of other primary malignancies, as well as a history of adjuvant radiotherapy or chemotherapy. Written consent to participate in the study was provided by every patient. The study protocol was approved by the Ethics Committee at Collegium Medicum in Bydgoszcz, Nicolaus Copernicus University in Torun (approval number: KB 1014/07). 

Tumor tissues obtained during surgery were routinely processed as formalin fixed and paraffin embedded (FFPE). DNA was isolated using deparaffinization of FFPE samples. Genomic DNA was extracted using Maxwell^®^ 16 FFPE Tissue LEV DNA Purification Kit. The DNA concentration was determined using the Plexor^®^ HY System. The methylation status of the *MGMT* promoter was tested using the MLPA probe mix prepared by MRC Holland (Salsa MS-MLPA Kit ME011 MMR), methylation-sensitive restriction enzyme, HhaI, and pyrosequencing using the *MGMT* Pyro Kit (QIAGEN) after DNA conversion and elution using the EpiTectBisulfite Kit. The relationships between *MGMT* promoter methylation, patient outcome, and the impact of clinical factors (tumor size, age, tumor location, and neurological status) on prognosis were evaluated.

### Statistical Analysis

Statistical analyses were performed using R and Google Sheets. Means (standard deviation (SD)) were used to describe normally and non-normally distributed data. The OS curves were plotted using the Kaplan–Meier technique and log-rank tests were applied to assess the difference in survival. OS was measured from the surgery date to the date of death or last follow-up. Levene’s test was used to assess the equality of variances. All comparisons between variables were made by means of the Mann–Whitney and Kruskal–Wallis nonparametric *t*-test. The Spearman-rank correlation coefficient was used to assess the relationship between variables. The findings were measured using 95% confidence intervals (95% CI), and a *p*-value of 0.05 was considered statistically significant.

## 3. Results

**Figure 1:**
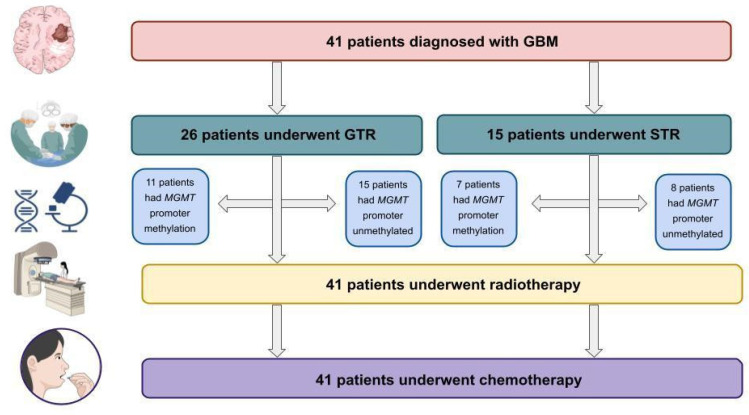
The flow chart of the study. GTR—Gross Total Resection; STR—Subtotal Resection.

Participants in the study included 41 patients who were newly diagnosed with glioblastoma and were treated at the 10th Military Research Hospital and Polyclinic in Bydgoszcz, Poland, between the years 2011 and 2014. All of the patients received surgical resection, which was then followed by radiation treatment and chemotherapy with alkylating drugs. The flow chart of the study is presented in Figure 1. The characteristics of the group being studied are detailed in Table 1.

Of the 41 newly diagnosed glioblastoma patients, 32 (78%) were men and 9 (22%) were women. The median age at the onset of disease was 53 years (interquartile range (IQR), 43.0–63.0). The most common locations for GBM were the temporal (61%), frontal (19.5%), and parietal lobes (14.6%), respectively. Eighteen patients (43.9%) had methylation of the promoter *MGMT* gene, and twenty-three patients had the gene promoter unmethylated (56.1%) (Table 2). The mean tumor volume was 38.1 cm^3^ ± 24.6. GTR was performed in 26 patients (63.4%) and subtotal (STR) in 15 cases. The preoperative KPS score was 90.0 ± 7 (median = 90). The majority of patients (68.3%) had an ASA score of one.

**Figure 2:**
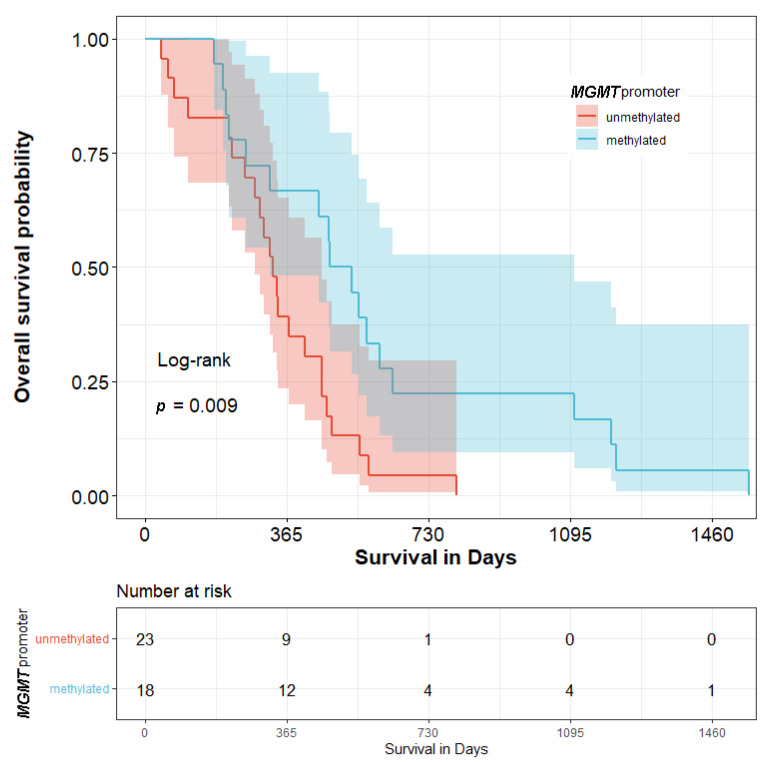
Kaplan–Meier curves of patients with primary glioblastoma—the relationship between *MGMT* methylation and overall survival among glioblastoma patients.

A significant difference between the *MGMT*-methylated and unmethylated groups in terms of median OS is demonstrated in Figure 2 (504 days vs. 329 days) (*p* < 0.05).

**Figure 3:**
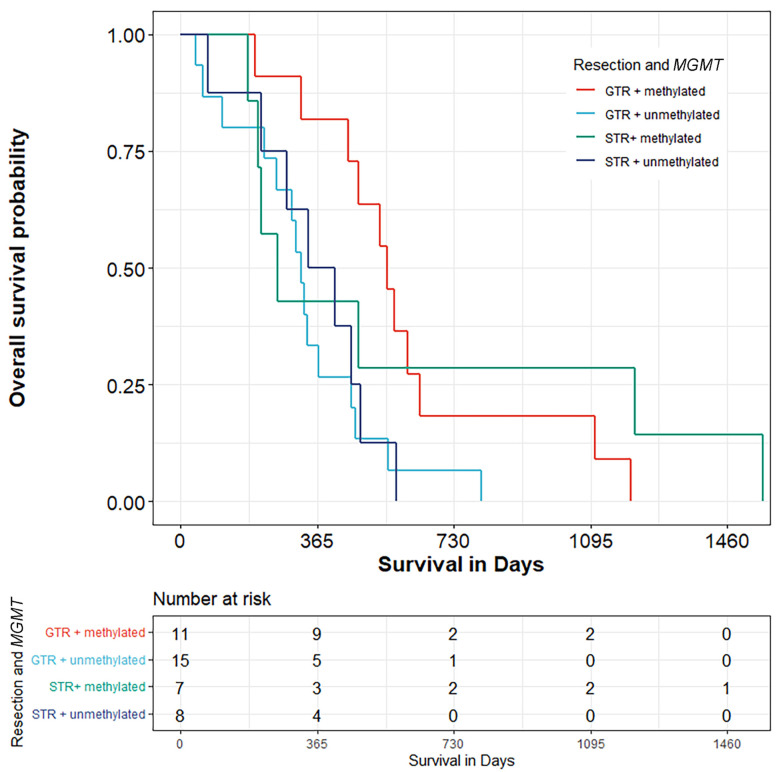
Kaplan–Meier curves of primary glioblastoma patients—association of gene methylation and extension of resection.

Among twenty-six cases that underwent GTR, eleven (42.3%) had *MGMT* promoter methylation, and fifteen of them did not. After GTR, patients with methylation had a median life duration of 551 days. Without methylation, the median life duration was 321 days. Seven patients had methylated *MGMT* promoter in a study group that underwent STR, with a median survival time of 260 days. Eight patients had unmethylated *MGMT* promoters, with a median survival time of 376.5 days, *p* = 0.05 (Figure 3). 

GTR + met vs. GTR + unmet.—< 0.05

GTR + met vs. STR + met.—*p* < 0.05

GTR + met vs. STR + unmet.—*p* < 0.05

In patients under 53 years old, those with *MGMT* methylation had significantly longer OS (639 days) in comparison to those without *MGMT* methylation (OS = 433.5 days; *p* = 0.046). However, a comparative survival analysis of patients without methylation did not demonstrate a substantial discrepancy (*p* > 0.05) between older and younger patients.

**Figure 4:**
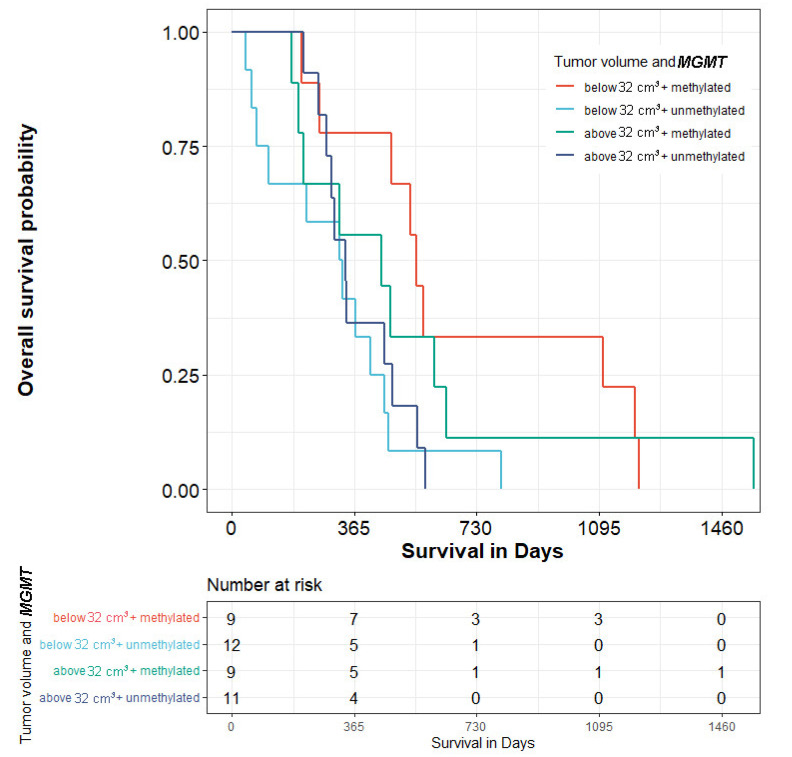
Kaplan–Meier curves of patients with primary glioblastoma—association of gene methylation, tumor size, and overall survival.

The median tumor size was 32 cm^3^. Among twenty-one patients with baseline tumor volume ≤32 cm^3^, ten (47.6%) had *MGMT* methylation, and eleven had no methylation. Patients with methylated *MGMT* had significantly longer median OS (560.5 days vs. 329 days; *p* < 0.05) than patients without methylation (Figure 4).

Size < 32 cm^3^ and met. *MGMT* gene vs. size < 32 cm^3^ and unmet. *MGMT* gene-*p* < 0.05

Size < 32 cm^3^ and met. *MGMT* gene vs. size > 32 cm^3^ and met. *MGMT* gene-*p* < 0.05

Size < 32 cm^3^ and met. *MGMT* gene vs. size > 32 cm^3^ and unmet. *MGMT* gene-*p* < 0.05

*MGMT*-methylated and unmethylated tumors did not significantly differ in terms of survival in patients with tumor volumes greater than 32 cm^3^. The location of the brain tumor was not substantially associated with OS.

## 4. Discussion

The molecular profile of malignant tumors is the foundation for modern management of primary brain tumors. Genetic characteristics have an essential role in clinical prognosis and selecting suitable treatment options. When evaluating the OS of patients with GBM, the presence of *MGMT* promoter gene methylation, the degree of tumor resection, the patient’s age, and the location of the tumor were all taken into account by the authors. The study revealed that GBM patients with *MGMT* gene promoter methylation responded more favorably to adjuvant therapy and, consequently, survived longer. 

*MGMT* status was the strongest predictor of prolonged survival in GBM patients receiving alkylating agent treatment [20,21,23,28]. Even in patients with nonresectable GBM, promoter methylation status played a crucial role in the survival prognosis [29]. Conversely, Jovanovic et al. showed that *MGMT* promoter methylation was not associated with the survival results of diffuse glioma patients [24]. In our study, 18 out of 41 patients had *MGMT* promoter methylation, which accounted for 43%. Zawlik et al. presented the prevalence of promoter methylation of *MGMT* in 371 cases with GBM in Switzerland, receiving a result of 44%, which was quite comparable to our study [22]. In a cohort study by Abhinav et al., the *MGMT* methylation ratio reached 59.6% [23]. 

According to the results of our study, younger age (less than 53) and GTR, which were plausible in smaller tumors (<32 cm^3^), are positive prognostic factors for OS. These findings are concordant with previous studies [30,31]. The median patient age in our study was 53 years old. This cut-off age was consistent with the findings of the population-based survey in Switzerland, where younger patients (<50 years) had a median survival time of 8.8 months. This was much longer than older patients (>80 years), who had a survival time of 1.6 months [32]. Furthermore, Jovanović et al. observed that in comparison to younger patients, people over the age of 50 had considerably poorer overall survival rates (median survival ranged from 7 to 19 months) [24], which is in accordance with our results. The OS in our younger study group was over twice as long (20 months with methylation and 14 months without methylation) as in the older group. In the study mentioned above, age was the most important univariate and multivariate prognostic factor [32]. Higher mortality for older individuals could be associated with co-morbidities and worse tolerance to surgery and adjuvant therapy [20]. Another possible mechanism associated with aging is the disruption of DNA methylation, characterized by hypomethylation across the whole genome, as well as methylation at particular promoters [33].

Patients in our study underwent surgical resection followed by combined radiotherapy and adjuvant temozolomide therapy, because the benefits of this kind of therapy last for five years [20]. Our study presented evidence that patients with baseline tumor volume ≤32 cm^3^ who had *MGMT* methylation lived significantly longer (median 560.5 days vs. 329 days without methylation). This can be attributed to more radical resection of the smaller tumors in contrast with tumors with a larger volume. In contrast, Wood et al. demonstrated that tumor size in high-grade glioma did not influence prognosis [34]. Several studies did not show a strong correlation between preoperative imaging of tumor size and OS, although the extent of resection was important [35,36].

Our research revealed that *MGMT* methylation is not only a favorable predictive biomarker for TMZ treatment, but it is also a positive prognosis factor. Numerous studies have indicated that *MGMT* methylation predicts prolonged OS [37,38,39]. Zhang et al. performed a meta-analysis on 29 studies that reported the influence of *MGMT* promoter methylation on OS. The combined hazard ratios were 0.67 (95% confidence interval [CI]: 0.58–0.78) in a univariate analysis of 15 studies and 0.49 (95% CI: 0.38–0.64) in a multivariate analysis of 14 studies [40]. In several trials, PFS was prolonged in individuals with methylated *MGMT* promoters who underwent TMZ treatment [18,41,42,43]. 

Nonetheless, there are indications that methylation of *MGMT* promoter is not a prognostic indicator in a subset of individuals. Nguyen et al. observed that *MGMT*-methylated patients had improved survival only when *TERT* promoter mutations were present [44]. In addition, Dahlrot et al. identified a correlation between *MGMT* methylation and OS beginning 9 months after diagnosis; however, there was no relation before this [45].

There are several possible explanations for the contradictory findings regarding *MGMT’s* prognostic value. First and foremost, there is strong evidence that the methylation status and the rate of protein production are inconsistent in terms of their relationship with patient prognosis [46,47]. Numerous studied have shown that *MGMT* promoter methylation and expression are negatively correlated. There are, nevertheless, constant indications of disagreement. Based on 52 studies, a meta-analysis found that the IHC and methylation-specific PCR (MSP) findings for malignancies were not closely correlated, suggesting that the two methods are not interchangeable [48]. When 76 glioblastoma samples were analyzed, there was no correlation between MSP methylation and *MGMT* protein expression (*p* = 0.903). Overall, 52.4% of tumors that were unmethylated and 41.2% that were methylated had low *MGMT* expression. There was a substantial correlation between *MGMT* methylation and patient survival, but there was no association between *MGMT* expression and survival [49]. Compared to IHC, pyrosequencing yielded a concordance rate of only 30.8% in another study [50]. In addition, when the analysis was expanded to a validation set using TCGA-GBM (The Cancer Genome Atlas—Glioblastoma Multiforme data, discrepancies were discovered in 46 of 209 samples (22%), which was comparable to the study population (19%) [51]. 

While promoter methylation is the major method for gene silencing, additional variables can also have an impact on the association between *MGMT* methylation, expression, and treatment effects. For example, the lack of connection between the results of the IHC examination with patient outcomes might be partially justified by test limitations.

### 4.1. MGMT Methylation Assessing Methods’ Limitations

Glioma methylation patterns are quite heterogeneous, and it is unclear which CpGs correlate most significantly with gene silencing. There are 97 CpG dinucleotides in the *MGMT* promoter sequence [52]. Notably, all methylation approaches sample just a small number of these compounds, often between 3 and 5. Although MSP has dominated *MGMT* methylation testing, there has been controversy as to whether it is the best and most appropriate method. The MSP typically examines 3–5 CpGs [53].

Pyrosequencing is a quantitative approach for measuring the methylation of the *MGMT* promoter. It typically analyzes a broader spectrum of CpGs than MSP and can analyze methylation levels at each CpG separately [53]. Pyrosequencing generates a quantitative measure of methylation and automatically calculates and reports the percentage of methylation for each CpG site in the sequence of study. Hence, this provides the detection of partially methylated CpG sites. Pyrosequencing has been demonstrated to be a more accurate method than MSP for assessing methylation status. However, it is more expensive and requires specialized equipment [54]. The EANO guidelines recommend the use of methylation-specific PCR, pyrosequencing, or methylation arrays [55]. In addition, Quillien et al. compared various evaluation methods of *MGMT* methylation and concluded that pyrosequencing had the highest reproducibility and sensitivity of the five techniques examined in this study [56]. Moreover, IHC is not recommended by ESMO to determine the *MGMT* promoter methylation status [55]. This is primarily due to variable thresholds for calling *MGMT* methylation positivity [57] and significant intra-observer diversity in *MGMT* methylation status assessments [58]. Moreover, contamination by non-neoplastic *MGMT*-expressing cells could result in false-positive tumor sample scoring [59].

While the interpretation of somatic mutations is unambiguous, assessing *MGMT* methylation status in routine clinical practice has been challenging. There is no well-established cut-off for *MGMT* promoter methylation status. Reifenberger et al. showed that patients treated with radiotherapy and chemotherapy or chemotherapy alone had a longer OS when pyrosequencing (PSQ) revealed highly methylated *MGMT* promoter alleles (>20–29%) [60]. Therefore, a strict PSQ cut-off at 10% might not fully reflect the clinical response to alkylating agents [30,43,61]. On the other hand, Zhao et al. reported that regardless of the PSQ cut-off for *MGMT* promoter methylation, methylated-positive patients were associated with improved HRs for OS and PFS [21]. Consensus is difficult to achieve due to differences in laboratory detection methods [62]. The cut-off values used in different studies are not always the same. When it comes to correlations, some researchers have found that differing cut-off values can have an impact on their own results. This lack of standardization impacts the evaluation of these tests’ association with patient outcomes and clinical value.

### 4.2. Mismatch Repair (MMR) Deficiency

TMZ cytotoxicity requires MMR activity. To avoid the lethal effects of TMZ, tumors that lack any of the four MMR proteins in particular (MLH1, MSH2, MSH6, and PMS2) are incapable of detecting the mispairing of O6-MeG and thymine [63]. Moreover, this may lead to the hypermutation phenotype being defined by a dramatic increase in the mutation rate. This phenomenon rarely occurs in newly diagnosed gliomas, but it is common in recurrent tumors after the use of alkylating agents This may partially explain the resistance observed in recurrent tumors regardless of the methylation status of the *MGMT* promoter. The best predictor of sensitivity to TMZ in patient-derived xenograft models of glioblastoma was found to be a combination of low *MGMT* activity and intact MMR [64].

### 4.3. MGMT Inhibitors

The fact that *MGMT* activity has a major effect on the susceptibility of tumor cells to O6-alkylating medicines has encouraged the search for techniques to reduce MGMT activity in tumor tissue in order to increase the sensitivity of tumor cells to these agents. In the following years, a significant number of O6-guanine derivatives and similar compounds were described and utilized to inactivate *MGMT* in a variety of experimental settings. Numerous tests demonstrated that O6-benzylguanine (O6-BG) or O6-bromothenylguanine (O6-BTG) was the most potent of these agents [9].

However, only one research trial (NCT01269424) involving O6-BG for the treatment of individuals with newly diagnosed glioblastoma multiforme is currently enrolling participants. The only completed phase 3 trial (NCT00017147) concluded that the addition of O6-BG to the conventional regimen of radiation and BCNU for the treatment of patients with newly diagnosed glioblastoma and gliosarcoma did not provide further benefit and instead increased toxicity [65]. Recent studies on *MGMT* inhibitors O6-BG and O6-BTG failed to increase the therapeutic response when combined with alkylating chemotherapeutics, highlighting the need for inhibitor targeting. Therefore, it would be highly desirable to develop methods to selectively target the MGMT-inactivating medication to the tumor.

### 4.4. Practical Application of MGMT Promoter Methylation Status

The methylation status of tumor-related genes demonstrates that they are important biomarkers not only in GBM, but also in other cancers, such as those of the Head and Neck [66]. As indicated above, *MGMT* promoter methylation status may alter the prognosis of primary GBM patients treated with TMZ. EANO recommends evaluating the *MGMT* promoter methylation status of elderly or frail patients with high-grade gliomas to determine whether temozolomide is required. However, in younger patients, *MGMT* promoter methylation status is not the main factor influencing the choice of systemic treatment. This is because currently, PVC (procarbazine, lomustine, and vincristine) therapy is one of the few alternatives that is very toxic, and the main factor influencing its selection is the high KPS [55]. However, the paradigm could soon be changed by the research carried out by Shaofang Wu and colleagues, which showed that PARylation of *MGMT* by PARP is necessary for the repair of the changes caused by TMZ, and that reduction in PARylation via a PARP inhibitor decreases *MGMT* function. This in turn, increases susceptibility to TMZ. Therefore, in the future, *MGMT* promoter methylation will be used as a predictive biomarker for the usage of PARP inhibitors [67]. Lastly, *MGMT* promoter methylation is utilized in clinical studies for risk stratification [7].

### 4.5. Limitations to This Study

There are several limitations of the present study. Our study was conducted on a relatively small group of patients treated in a single institution, and we did not include progression-free survival. Nonetheless, this cohort represents a larger Polish population of patients with GBM and reflects the true prevalence of *MGMT* gene promoter methylation in this population. Due to the fact that the study started before the WHO CNS 2016 and had a long follow-up period, we did not assess the mutation in *IDH*, which is currently also of prognostic significance.

## 5. Conclusions

In our study, *MGMT* promoter methylation was a favorable prognostic factor, with the longest survival in younger patients at the time of diagnosis (≤50 years), small tumor (≤32 cm^3^), and GTR. Epigenetic silencing of the *MGMT* DNA-repair gene by promoter methylation has been associated with longer survival in patients with GBM who receive alkylating agents. *MGMT* promoter methylation is not an ideal prognostic factor. More research is needed to assess its relationship with *MGMT* expression, MMR, or *MGMT* gene body methylation to fully understand its prognostic potential. 

## Figures and Tables

**Table 1:** Characteristics of Patients.

		N	%
Age, mean ± SD		53	12
Sex	Female	9	22%
	Male	32	78%
Tumor Volume [cm^3^], mean ± SD		38.1	24.6
The Extent of Resection			
	GTR	26	63.4%
	STR	15	36.6%
KPS, mean ± SD		90	7
*MGMT* Methylation Status			
	Unmethylated	23	56.1%
	Methylated	18	43.9%
ASA score			
	1	28	68.3%
	2	10	24.4%
	3	3	7.3%
Location			
	Frontal	8	19.5%
	Temporal	25	61%
	Parietal	6	14.6%
	Occipital	1	2.4%
	Multifocal	1	2.4%

Note: GTR—Gross Total Resection; STR—Subtotal Resection; KPS—Karnofsky Performance Status; ASA score—American Society of Anesthesiologists score; *MGMT*—O^6^-methylguanine—DNA methyltransferase.

**Table 2:** The association between methylation of the *MGMT* promoter and patient characteristics.

Variable		Number of Patients	*MGMT* Methylation
Sex	Female	9	5
	Male	32	13
Age	≤53	21	9
	>53	20	9
Tumor size (cm^3^)	≤32	21	10
	>32	20	8
Resection rate	Gross total resection	26	11
	Subtotal resection	15	7

Note: *MGMT*-O^6^-methylguanine-DNA methyltransferase.

## Data Availability

All data relevant to this study are reported within the manuscript.

## References

[1] Louis D.N., Ohgaki H., Wiestler O.D., Cavenee W.K., Burger P.C., Jouvet A., Scheithauer B.W., Kleihues P. (2007). The 2007 WHO Classification of Tumours of the Central Nervous System. Acta Neuropathol..

[2] Müller Bark J., Kulasinghe A., Chua B., Day B.W., Punyadeera C. (2020). Circulating Biomarkers in Patients with Glioblastoma. Br. J. Cancer.

[3] Furtak J., Kwiatkowski A., Śledzińska P., Bebyn M., Krajewski S., Szylberg T., Birski M., Druszcz A., Krystkiewicz K., Gasiński P. (2022). Survival after Reoperation for Recurrent Glioblastoma Multiforme: A Prospective Study. Surg. Oncol..

[4] Miranda A., Blanco-Prieto M., Sousa J., Pais A., Vitorino C. (2017). Breaching Barriers in Glioblastoma. Part I: Molecular Pathways and Novel Treatment Approaches. Int. J. Pharm..

[5] Li H., Li J., Cheng G., Zhang J., Li X. (2016). IDH Mutation and MGMT Promoter Methylation Are Associated with the Pseudoprogression and Improved Prognosis of Glioblastoma Multiforme Patients Who Have Undergone Concurrent and Adjuvant Temozolomide-Based Chemoradiotherapy. Clin. Neurol. Neurosurg..

[6] Esteller M., Garcia-Foncillas J., Andion E., Goodman S.N., Hidalgo O.F., Vanaclocha V., Baylin S.B., Herman J.G. (2000). Inactivation of the DNA-Repair Gene MGMT and the Clinical Response of Gliomas to Alkylating Agents. N. Engl. J. Med..

[7] Hegi M.E., Diserens A.-C., Gorlia T., Hamou M.-F., de Tribolet N., Weller M., Kros J.M., Hainfellner J.A., Mason W., Mariani L. (2005). MGMT Gene Silencing and Benefit from Temozolomide in Glioblastoma. N. Engl. J. Med..

[8] Villano J.L., Seery T.E., Bressler L.R. (2009). Temozolomide in Malignant Gliomas: Current Use and Future Targets. Cancer Chemother. Pharmacol..

[9] Kaina B., Christmann M. (2019). DNA Repair in Personalized Brain Cancer Therapy with Temozolomide and Nitrosoureas. DNA Repair.

[10] Silber J.R., Blank A., Bobola M.S., Mueller B.A., Kolstoe D.D., Ojemann G.A., Berger M.S. (1996). Lack of the DNA Repair Protein O6-Methylguanine-DNA Methyltransferase in Histologically Normal Brain Adjacent to Primary Human Brain Tumors. Proc. Natl. Acad. Sci. USA.

[11] Bobola M.S., Alnoor M., Chen J.Y.-S., Kolstoe D.D., Silbergeld D.L., Rostomily R.C., Blank A., Chamberlain M.C., Silber J.R. (2015). O6-Methylguanine-DNA Methyltransferase Activity Is Associated with Response to Alkylating Agent Therapy and with MGMT Promoter Methylation in Glioblastoma and Anaplastic Glioma. BBA Clin..

[12] Yuan G., Niu L., Zhang Y., Wang X., Ma K., Yin H., Dai J., Zhou W., Pan Y. (2017). Defining Optimal Cutoff Value of MGMT Promoter Methylation by ROC Analysis for Clinical Setting in Glioblastoma Patients. J. Neurooncol..

[13] Binabaj M.M., Bahrami A., ShahidSales S., Joodi M., Joudi Mashhad M., Hassanian S.M., Anvari K., Avan A. (2018). The Prognostic Value of MGMT Promoter Methylation in Glioblastoma: A Meta-Analysis of Clinical Trials. J. Cell. Physiol..

[14] Cabrini G., Fabbri E., Lo Nigro C., Dechecchi M.C., Gambari R. (2015). Regulation of Expression of O6-Methylguanine-DNA Methyltransferase and the Treatment of Glioblastoma (Review). Int. J. Oncol..

[15] Verbeek B., Southgate T.D., Gilham D.E., Margison G.P. (2008). O6-Methylguanine-DNA Methyltransferase Inactivation and Chemotherapy. Br. Med. Bull..

[16] Wiewrodt D., Nagel G., Dreimüller N., Hundsberger T., Perneczky A., Kaina B. (2008). MGMT in Primary and Recurrent Human Glioblastomas after Radiation and Chemotherapy and Comparison with P53 Status and Clinical Outcome. Int. J. Cancer.

[17] Chiang J., Harreld J.H., Tinkle C.L., Moreira D.C., Li X., Acharya S., Qaddoumi I., Ellison D.W. (2019). A Single-Center Study of the Clinicopathologic Correlates of Gliomas with a MYB or MYBL1 Alteration. Acta Neuropathol..

[18] Yang P., Zhang W., Wang Y., Peng X., Chen B., Qiu X., Li G., Li S., Wu C., Yao K. (2015). IDH Mutation and MGMT Promoter Methylation in Glioblastoma: Results of a Prospective Registry. Oncotarget.

[19] Roszkowski K., Furtak J., Zurawski B., Szylberg T., Lewandowska M.A. (2016). Potential Role of Methylation Marker in Glioma Supporting Clinical Decisions. Int. J. Mol. Sci..

[20] Stupp R., Hegi M.E., Mason W.P., van den Bent M.J., Taphoorn M.J.B., Janzer R.C., Ludwin S.K., Allgeier A., Fisher B., Belanger K. (2009). Effects of Radiotherapy with Concomitant and Adjuvant Temozolomide versus Radiotherapy Alone on Survival in Glioblastoma in a Randomised Phase III Study: 5-Year Analysis of the EORTC-NCIC Trial. Lancet Oncol..

[21] Zhao H., Wang S., Song C., Zha Y., Li L. (2016). The Prognostic Value of MGMT Promoter Status by Pyrosequencing Assay for Glioblastoma Patients’ Survival: A Meta-Analysis. World J. Surg. Oncol..

[22] Zawlik I., Vaccarella S., Kita D., Mittelbronn M., Franceschi S., Ohgaki H. (2009). Promoter Methylation and Polymorphisms of the MGMT Gene in Glioblastomas: A Population-Based Study. Neuroepidemiology.

[23] Abhinav K., Aquilina K., Gbejuade H., La M., Hopkins K., Iyer V. (2013). A Pilot Study of Glioblastoma Multiforme in Elderly Patients: Treatments, O-6-Methylguanine-DNA Methyltransferase (MGMT) Methylation Status and Survival. Clin. Neurol. Neurosurg..

[24] Jovanović N., Mitrović T., Cvetković V.J., Tošić S., Vitorović J., Stamenković S., Nikolov V., Kostić A., Vidović N., Jevtović-Stoimenov T. (2019). Prognostic Significance of MGMT Promoter Methylation in Diffuse Glioma Patients. Biotechnol. Biotechnol. Equip..

[25] Śledzińska P., Bebyn M.G., Furtak J., Kowalewski J., Lewandowska M.A. (2021). Prognostic and Predictive Biomarkers in Gliomas. Int. J. Mol. Sci..

[26] Péus D., Newcomb N., Hofer S. (2013). Appraisal of the Karnofsky Performance Status and Proposal of a Simple Algorithmic System for Its Evaluation. BMC Med. Inform. Decis. Mak..

[27] Park J.-H., Kim D.-H., Kim B.-R., Kim Y.-W. (2018). The American Society of Anesthesiologists Score Influences on Postoperative Complications and Total Hospital Charges after Laparoscopic Colorectal Cancer Surgery. Medicine.

[28] Thon N., Kreth S., Kreth F.-W. (2013). Personalized Treatment Strategies in Glioblastoma: MGMT Promoter Methylation Status. OncoTargets Ther..

[29] Iaccarino C., Orlandi E., Ruggeri F., Nicoli D., Torricelli F., Maggi M., Cerasti D., Pisanello A., Pedrazzi G., Froio E. (2015). Prognostic Value of MGMT Promoter Status in Non-Resectable Glioblastoma after Adjuvant Therapy. Clin. Neurol. Neurosurg..

[30] Radke J., Koch A., Pritsch F., Schumann E., Misch M., Hempt C., Lenz K., Löbel F., Paschereit F., Heppner F.L. (2019). Predictive MGMT Status in a Homogeneous Cohort of IDH Wildtype Glioblastoma Patients. Acta Neuropathol. Commun..

[31] Dunn J., Baborie A., Alam F., Joyce K., Moxham M., Sibson R., Crooks D., Husband D., Shenoy A., Brodbelt A. (2009). Extent of MGMT Promoter Methylation Correlates with Outcome in Glioblastomas given Temozolomide and Radiotherapy. Br. J. Cancer.

[32] Ohgaki H., Dessen P., Jourde B., Horstmann S., Nishikawa T., Di Patre P.-L., Burkhard C., Schüler D., Probst-Hensch N.M., Maiorka P.C. (2004). Genetic Pathways to Glioblastoma: A Population-Based Study. Cancer Res..

[33] Yang J., Yu L., Gaiteri C., Srivastava G.P., Chibnik L.B., Leurgans S.E., Schneider J.A., Meissner A., De Jager P.L., Bennett D.A. (2015). Association of DNA Methylation in the Brain with Age in Older Persons Is Confounded by Common Neuropathologies. Int. J. Biochem. Cell Biol..

[34] Wood J.R., Green S.B., Shapiro W.R. (1988). The Prognostic Importance of Tumor Size in Malignant Gliomas: A Computed Tomographic Scan Study by the Brain Tumor Cooperative Group. J. Clin. Oncol. Off. J. Am. Soc. Clin. Oncol..

[35] Lacroix M., Abi-Said D., Fourney D.R., Gokaslan Z.L., Shi W., DeMonte F., Lang F.F., McCutcheon I.E., Hassenbusch S.J., Holland E. (2001). A Multivariate Analysis of 416 Patients with Glioblastoma Multiforme: Prognosis, Extent of Resection, and Survival. J. Neurosurg..

[36] McGirt M.J., Chaichana K.L., Gathinji M., Attenello F.J., Than K., Olivi A., Weingart J.D., Brem H., Quiñones-Hinojosa A.R. (2009). Independent Association of Extent of Resection with Survival in Patients with Malignant Brain Astrocytoma. J. Neurosurg..

[37] Brat D.J., Verhaak R.G.W., Aldape K.D., Yung W.K.A., Salama S.R., Cooper L.A.D., Rheinbay E., Miller C.R., Vitucci M., Cancer Genome Atlas Research Network (2015). Comprehensive, Integrative Genomic Analysis of Diffuse Lower-Grade Gliomas. N. Engl. J. Med..

[38] Shu C., Wang Q., Yan X., Wang J. (2018). The TERT Promoter Mutation Status and MGMT Promoter Methylation Status, Combined with Dichotomized MRI-Derived and Clinical Features, Predict Adult Primary Glioblastoma Survival. Cancer Med..

[39] (2010). Reis Prognostic Value of MGMT Promoter Methylation in Glioblastoma Patients Treated with Temozolomide-Based Chemoradiation: A Portuguese Multicentre Study. Oncol. Rep..

[40] Zhang K., Wang X., Zhou B., Zhang L. (2013). The Prognostic Value of MGMT Promoter Methylation in Glioblastoma Multiforme: A Meta-Analysis. Fam. Cancer.

[41] SongTao Q., Lei Y., Si G., YanQing D., HuiXia H., XueLin Z., LanXiao W., Fei Y. (2012). IDH Mutations Predict Longer Survival and Response to Temozolomide in Secondary Glioblastoma. Cancer Sci..

[42] Li J., Liang R., Song C., Xiang Y., Liu Y. (2020). Prognostic and Clinicopathological Significance of Long Non-Coding RNA in Glioma. Neurosurg. Rev..

[43] Chai R., Li G., Liu Y., Zhang K., Zhao Z., Wu F., Chang Y., Pang B., Li J., Li Y. (2021). Predictive Value of MGMT Promoter Methylation on the Survival of TMZ Treated IDH-Mutant Glioblastoma. Cancer Biol. Med..

[44] Nguyen H.N., Lie A., Li T., Chowdhury R., Liu F., Ozer B., Wei B., Green R.M., Ellingson B.M., Wang H. (2017). Human TERT Promoter Mutation Enables Survival Advantage from MGMT Promoter Methylation in IDH1 Wild-Type Primary Glioblastoma Treated by Standard Chemoradiotherapy. Neuro-Oncology.

[45] Dahlrot R.H., Larsen P., Boldt H.B., Kreutzfeldt M.S., Hansen S., Hjelmborg J.B., Kristensen B.W. (2019). Posttreatment Effect of MGMT Methylation Level on Glioblastoma Survival. J. Neuropathol. Exp. Neurol..

[46] Uno M., Oba-Shinjo S.M., Camargo A.A., Moura R.P., de Aguiar P.H., Cabrera H.N., Begnami M., Rosemberg S., Teixeira M.J., Marie S.K.N. (2011). Correlation of MGMT Promoter Methylation Status with Gene and Protein Expression Levels in Glioblastoma. Clinics.

[47] Lalezari S., Chou A.P., Tran A., Solis O.E., Khanlou N., Chen W., Li S., Carrillo J.A., Chowdhury R., Selfridge J. (2013). Combined Analysis of O6-Methylguanine-DNA Methyltransferase Protein Expression and Promoter Methylation Provides Optimized Prognostication of Glioblastoma Outcome. Neuro-Oncology.

[48] Brell M., Ibáñez J., Tortosa A. (2011). O6-Methylguanine-DNA Methyltransferase Protein Expression by Immunohistochemistry in Brain and Non-Brain Systemic Tumours: Systematic Review and Meta-Analysis of Correlation with Methylation-Specific Polymerase Chain Reaction. BMC Cancer.

[49] Melguizo C., Prados J., González B., Ortiz R., Concha A., Alvarez P.J., Madeddu R., Perazzoli G., Oliver J.A., López R. (2012). MGMT Promoter Methylation Status and MGMT and CD133 Immunohistochemical Expression as Prognostic Markers in Glioblastoma Patients Treated with Temozolomide plus Radiotherapy. J. Transl. Med..

[50] Wang L., Li Z., Liu C., Chen L., Liu L., Hu Z., Zhao L., Lu D., Teng L. (2017). Comparative Assessment of Three Methods to Analyze MGMT Methylation Status in a Series of 350 Gliomas and Gangliogliomas. Pathol. Res. Pract..

[51] Kreth S., Thon N., Eigenbrod S., Lutz J., Ledderose C., Egensperger R., Tonn J.C., Kretzschmar H.A., Hinske L.C., Kreth F.W. (2011). O-Methylguanine-DNA Methyltransferase (MGMT) MRNA Expression Predicts Outcome in Malignant Glioma Independent of MGMT Promoter Methylation. PLoS ONE.

[52] Riemenschneider M.J., Hegi M.E., Reifenberger G. (2010). MGMT Promoter Methylation in Malignant Gliomas. Target. Oncol..

[53] Weller M., Stupp R., Reifenberger G., Brandes A.A., van den Bent M.J., Wick W., Hegi M.E. (2010). MGMT Promoter Methylation in Malignant Gliomas: Ready for Personalized Medicine?. Nat. Rev. Neurol..

[54] Christians A., Hartmann C., Benner A., Meyer J., von Deimling A., Weller M., Wick W., Weiler M. (2012). Prognostic Value of Three Different Methods of MGMT Promoter Methylation Analysis in a Prospective Trial on Newly Diagnosed Glioblastoma. PLoS ONE.

[55] Weller M., van den Bent M., Preusser M., Le Rhun E., Tonn J.C., Minniti G., Bendszus M., Balana C., Chinot O., Dirven L. (2021). EANO Guidelines on the Diagnosis and Treatment of Diffuse Gliomas of Adulthood. Nat. Rev. Clin. Oncol..

[56] Quillien V., Lavenu A., Karayan-Tapon L., Carpentier C., Labussière M., Lesimple T., Chinot O., Wager M., Honnorat J., Saikali S. (2012). Comparative Assessment of 5 Methods (Methylation-Specific Polymerase Chain Reaction, MethyLight, Pyrosequencing, Methylation-Sensitive High-Resolution Melting, and Immunohistochemistry) to Analyze O6-Methylguanine-DNA-Methyltranferase in a Series of 100 Glioblastoma Patients. Cancer.

[57] Mason S., McDonald K. (2012). MGMT Testing for Glioma in Clinical Laboratories: Discordance with Methylation Analyses Prevents the Implementation of Routine Immunohistochemistry. J. Cancer Res. Clin. Oncol..

[58] Preusser M., Charles Janzer R., Felsberg J., Reifenberger G., Hamou M.-F., Diserens A.-C., Stupp R., Gorlia T., Marosi C., Heinzl H. (2008). Anti-O6-Methylguanine-Methyltransferase (MGMT) Immunohistochemistry in Glioblastoma Multiforme: Observer Variability and Lack of Association with Patient Survival Impede Its Use as Clinical Biomarker. Brain Pathol..

[59] Liu L., Spiro T.P., Qin X., Majka S., Haaga J., Schupp J., Willson J.K., Gerson S.L. (2001). Differential Degradation Rates of Inactivated Alkyltransferase in Blood Mononuclear Cells and Tumors of Patients after Treatment with O(6)-Benzylguanine. Clin. Cancer Res. Off. J. Am. Assoc. Cancer Res..

[60] Reifenberger G., Hentschel B., Felsberg J., Schackert G., Simon M., Schnell O., Westphal M., Wick W., Pietsch T., Loeffler M. (2012). Predictive Impact of MGMT Promoter Methylation in Glioblastoma of the Elderly. Int. J. Cancer.

[61] Gurrieri L., De Carlo E., Gerratana L., De Maglio G., Macerelli M., Pisa F.E., Masiero E., Aprile G., Follador A., Puglisi F. (2018). MGMT Pyrosequencing-Based Cut-off Methylation Level and Clinical Outcome in Patients with Glioblastoma Multiforme. Future Oncol..

[62] Mansouri A., Hachem L.D., Mansouri S., Nassiri F., Laperriere N.J., Xia D., Lindeman N.I., Wen P.Y., Chakravarti A., Mehta M.P. (2019). MGMT Promoter Methylation Status Testing to Guide Therapy for Glioblastoma: Refining the Approach Based on Emerging Evidence and Current Challenges. Neuro-Oncology.

[63] Jiapaer S., Furuta T., Tanaka S., Kitabayashi T., Nakada M. (2018). Potential Strategies Overcoming the Temozolomide Resistance for Glioblastoma. Neurol. Med. Chir..

[64] Nagel Z.D., Kitange G.J., Gupta S.K., Joughin B.A., Chaim I.A., Mazzucato P., Lauffenburger D.A., Sarkaria J.N., Samson L.D. (2017). DNA Repair Capacity in Multiple Pathways Predicts Chemoresistance in Glioblastoma Multiforme. Cancer Res..

[65] Blumenthal D.T., Rankin C., Stelzer K.J., Spence A.M., Sloan A.E., Moore D.F., Padula G.D.A., Schulman S.B., Wade M.L., Rushing E.J. (2015). A Phase III Study of Radiation Therapy (RT) and O6-Benzylguanine + BCNU versus RT and BCNU Alone and Methylation Status in Newly Diagnosed Glioblastoma and Gliosarcoma: Southwest Oncology Group (SWOG) Study S0001. Int. J. Clin. Oncol..

[66] Misawa K., Mochizuki D., Imai A., Mima M., Misawa Y., Mineta H. (2018). Analysis of Site-Specific Methylation of Tumor-Related Genes in Head and Neck Cancer: Potential Utility as Biomarkers for Prognosis. Cancers.

[67] Wu S., Li X., Gao F., de Groot J.F., Koul D., Yung W.K.A. (2021). PARP-Mediated PARylation of MGMT Is Critical to Promote Repair of Temozolomide-Induced O6-Methylguanine DNA Damage in Glioblastoma. Neuro-Oncology.

